# Supplementary material for: Inhibition of Wdr5 Attenuates Ang-II-Induced Fibroblast-to-Myofibroblast Transition in Cardiac Fibrosis by Regulating Mdm2/P53/P21 Pathway
Source: Biomolecules. 2022 Oct 27;12(11):1574. doi: 10.3390/biom12111574 (PMC9687631; doi:10.3390/biom12111574)
Supplement: Supplementary file 1 [file biomolecules-12-01574-s001.zip › biomolecules-1898695-supplementary.pdf]

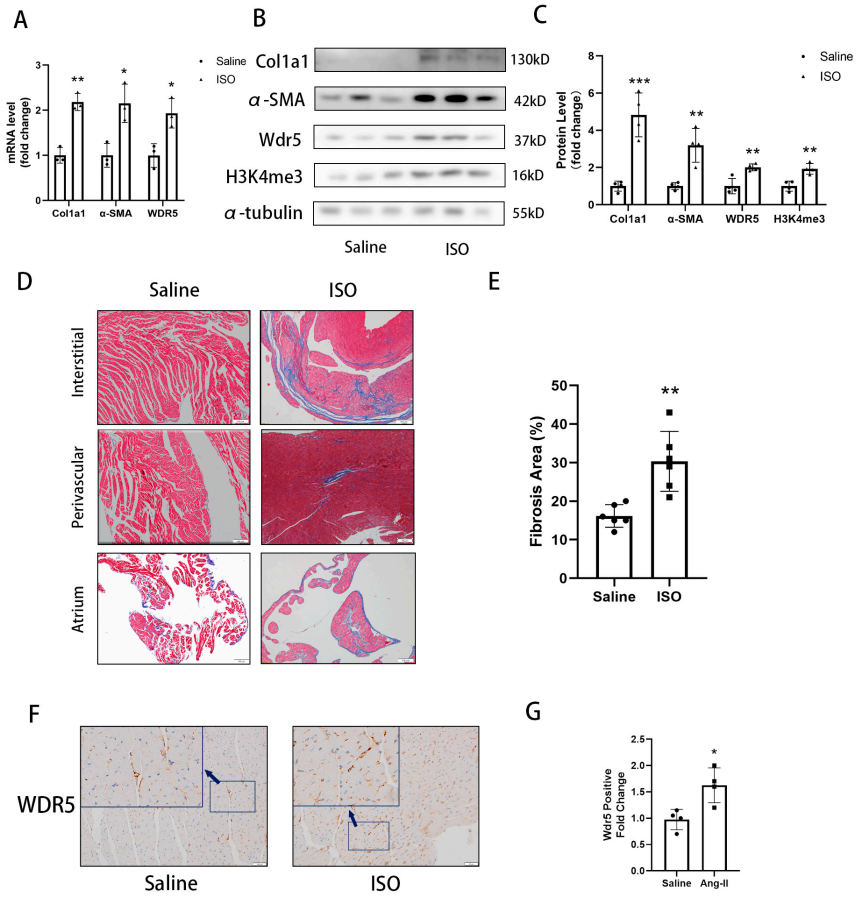

Figure S1 WDR5 expression were elevated in ISO-induced cardiac fibrosis models
A Col1a1, α-SMA, and WDR5 mRNA levels were significantly elevated in ISO-infused animal models. **B** Col1a1, α-SMA, WDR5, and H3K4me3 protein levels were significantly increased in ISO-infused animal compared with saline group (n=4 in each group). **C** is the quantitative statistic of E graph. **D** Masson's trichrome staining showed increased fibrosis after 14 days of ISO injection. **F** is the quantitative statistic of fibrosis areas in D (n=6 in each group). **F** IHC results indicated that WDR5 proteins were upregulated in ISO injected animals（n=4）. **G** is the quantitative analysis of F. *p<.05, **p<.01, ***p<.001, vs saline.

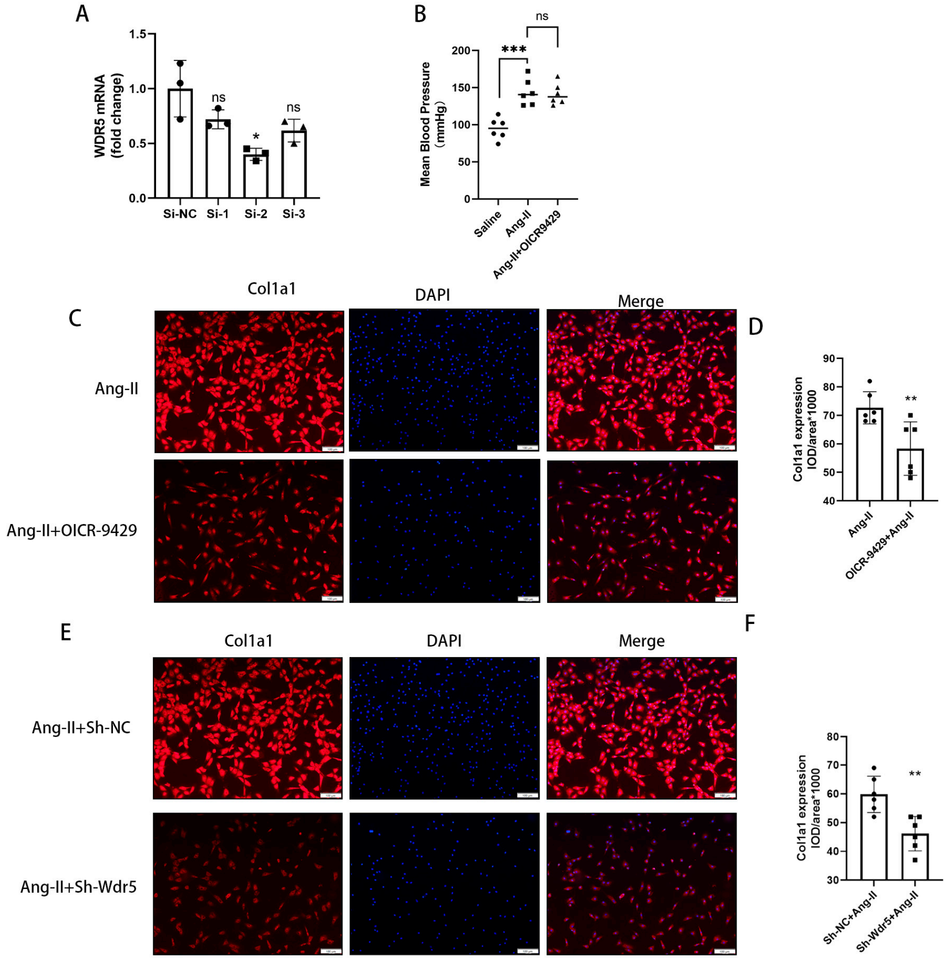

Figure S2 Inhibition of Wdr5 reduced the expression of Col1a1 in NRCFs

A qPCR showed knockdown efficacy of different Sh-Wdr5 hairpin sequence. **B** Application of OICR9429 didn't affect the mean blood pressure level of mice. **C** Immunofluorescence analysis of Col1a1 demonstrated inhibited Col1a1 by OICR-9429 (red, α-SMA; blue, DAPI) and **D** is the quantitative statics of graph C (n=6 in each group, >3 fields in each sample). **E** Immunofluorescence analysis of Col1a1 demonstrated inhibited Col1a1 expression by knocking down Wdr5 (red, α-SMA; blue, DAPI) and **F** is the quantitative statics of graph E (n=6 in each group, >3 fields in each sample).

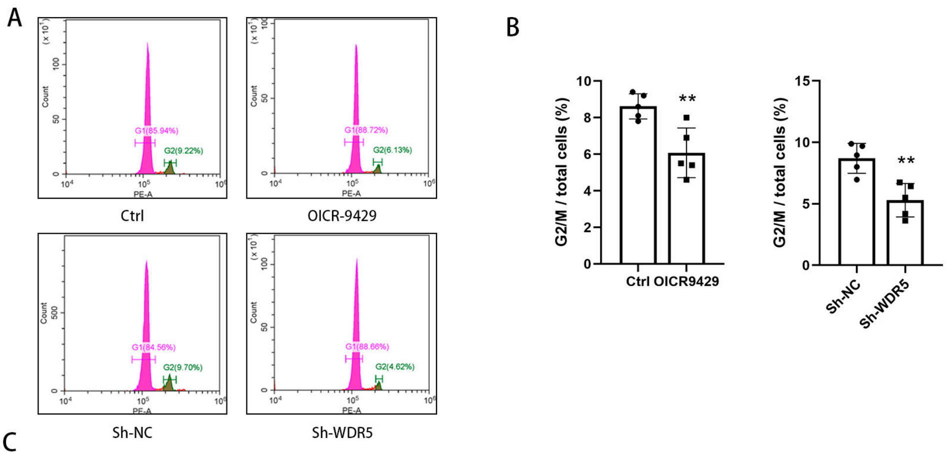

C

MDM2:

Amplicon A F: TTTTCAATCACCCACGCCAC R: ACATAGAGTGCAGTTGCCCA

Amplicon B F: GGCGTGGGTGATTGAAAACC R: CTGAGGAGATGGTTCCGTGG

Amplicon C F: CCACTTCCTCCTCGCTCAAG R: CGCCTCCTGAACCAATAGGG

P53:

Amplicon A F: GGAGTTGGTCATCTCCACCG R: CTCCGGATAGCCTCCCTTCT

Amplicon B F: CTGGGATTGGGACTTTCCCC R: ACCCCCTAACTGTAGTCGCT

P21

Amplicon A F: AGTTCGGGCAAAACCAAGGA R: ACAGAGTCTCTCAGGGGTCC

Amplicon B F: GACCTGAGGAGAGCCAACTG R: GTTAGCAGGAACTCGGGCTT

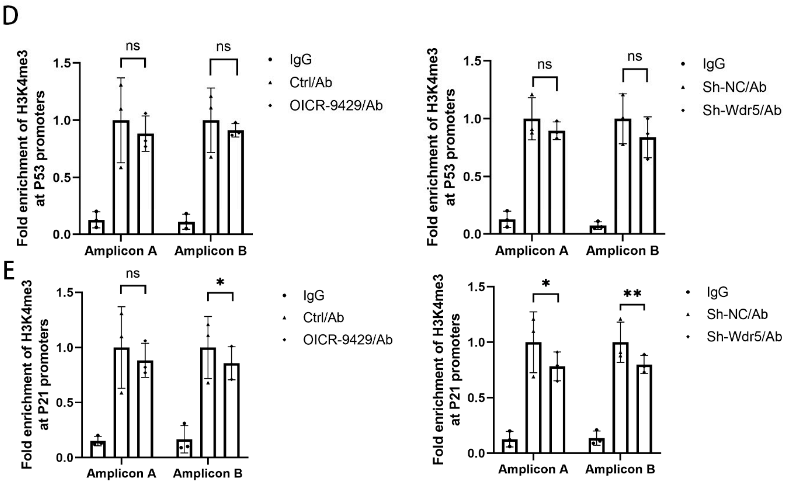

Figure S3 Inhibition of Wdr5 didn't affect H3K4me3 enrichment at promoters of *p53* and *p21*
A Flow CytoMetry analysis indicated G1 cell cycle arrested after pharmacology and genetic inhibition of Wdr5 and **B** is the quantitative statics of graph A. **C** Primer sequence of different Amplicons in Cut&Tag-qPCR. **D** and **E** Inhibition of Wdr5 had no effect on the H3K4me3 enrichment at *Mdm2* promoters.

Table S1 Sequences of primers for genes analyzed by qRT-PCR (m-mouse, R-rat).

Genes	Forward Primers	Backward Primers
Col1a1(m)	GCTCCTCTTAGGGGCCACT	CCACGTCTCACCATTGGGG
α-SMA(m)	GTCCCAGACATCAGGGAGTAA	TCGGATACTTCAGCGTCAGGA
Wdr5(m)	CCAGTCTCAGCCGTTCATTTC	GTGTCCCAGATTCGGCAGA
Gapdh(m)	AATGGATTTGGACGCATTGGT	TTTGCACTGGTACGTGTTGAT

Col1a1(R)	GGAGAGAGCATGACCGATGG	AAGTTCCGGTGTGACTCGTG
α-SMA(R)	CATCCGACCTTGCTAACGGA	AGTCCAGCACAATACCAGTTGT
Wdr5(R)	CCAGTCTCAGCCGTTCATTTC	GTGTCCCAGATTCGGCAGA
MDM2(R)	CCGAGCGAAATGGTCTCTCA	GGTATTGCACATTGGCCTGG
P53(R)	GGCAGACTTTTCGCCACAG	CAGGCACAAACACGAACCTC
P21(R)	TGTGATATGTACCAGCCACAGG	CAGACGTAGTTGCCCTCCAG
Gapdh(R)	GCCGCATCTTCTTGTGCAG	GGGTTTCCCGTTGATGACCA
